# Pain-Related Fear—Dissociable Neural Sources of Different Fear Constructs

**DOI:** 10.1523/ENEURO.0107-18.2018

**Published:** 2019-01-03

**Authors:** Michael Lukas Meier, Andrea Vrana, Barry Kim Humphreys, Erich Seifritz, Philipp Stämpfli, Petra Schweinhardt

**Affiliations:** 1 Integrative Spinal Research, Department of Chiropractic Medicine, Balgrist University Hospital, 8008 Zurich, Switzerland; 2 Department of Psychiatry, Psychotherapy and Psychosomatics, Hospital of Psychiatry, University of Zurich, 8006 Zurich, Switzerland; 3 MR-Center of the Psychiatric Hospital, University of Zurich, 8006 Zurich, Switzerland; 4 Alan Edwards Center for Research on Pain, McGill University, Montreal, Quebec, Canada

**Keywords:** amygdala, chronic pain, fear network, low back pain, machine learning, multivariate analysis

## Abstract

Fear of pain demonstrates significant prognostic value regarding the development of persistent musculoskeletal pain and disability. Its assessment often relies on self-report measures of pain-related fear by a variety of questionnaires. However, based either on “fear of movement/(re)injury/kinesiophobia,” “fear avoidance beliefs,” or “pain anxiety,” pain-related fear constructs plausibly differ while it is unclear how specific the questionnaires are in assessing these different constructs. Furthermore, the relationship of pain-related fear to other anxiety measures such as state or trait anxiety remains ambiguous. Advances in neuroimaging such as machine learning on brain activity patterns recorded by functional magnetic resonance imaging might help to dissect commonalities or differences across pain-related fear constructs. We applied a pattern regression approach in 20 human patients with nonspecific chronic low back pain to reveal predictive relationships between fear-related neural pattern information and different pain-related fear questionnaires. More specifically, the applied multiple kernel learning approach allowed the generation of models to predict the questionnaire scores based on a hierarchical ranking of fear-related neural patterns induced by viewing videos of activities potentially harmful for the back. We sought to find evidence for or against overlapping pain-related fear constructs by comparing the questionnaire prediction models according to their predictive abilities and associated neural contributors. By demonstrating evidence of nonoverlapping neural predictors within fear-processing regions, the results underpin the diversity of pain-related fear constructs. This neuroscientific approach might ultimately help to further understand and dissect psychological pain-related fear constructs.

## Significance Statement

Pain-related fear, often assessed through self-reports such as questionnaires, has shown prognostic value and clinical utility for a variety of musculoskeletal pain disorders. However, it remains difficult to determine a common underlying construct of pain-related fear due to several proposed constructs among questionnaires. The current study describes a novel neuroscientific approach using machine learning of neural patterns within the fear circuit of chronic low back pain patients that has the potential to identify neural commonalities or differences among the various constructs. Ultimately, this approach might afford a deeper understanding of the suggested constructs and might be also applied to other domains where ambiguity exists between different psychological constructs.

## Introduction

Self-report measures of emotional states are paramount for behavioral neuroscience by enabling the understanding of brain response patterns (Shrout et al., 2018). However, the validity of self-reports is limited (Choi and Pak, 2005), probably also because often overlapping psychological constructs are assessed, illustrated by the fact that various questionnaires attempt to assess related constructs. One example is pain-related fear (PRF), which is a major explanatory variable of disability in patients with persistent musculoskeletal pain (Crombez et al., 1999; Vlaeyen and Linton, 2000; Vlaeyen et al., 2016). For the assessment of PRF, various questionnaires exist based on potentially different constructs such as fear of movement/injury and reinjury/kinesiophobia, fear avoidance beliefs, or pain anxiety. There is an open debate on what their scores reflect on the fear–anxiety spectrum (Lundberg et al., 2011; Caneiro et al., 2017). Fear represents a reaction to an imminent threat, preparing the individual for “fight-flight-freeze,” whereas anxiety is described as being more diffuse (e.g., cognitions about a future threat; LeDoux and Pine, 2016; Kreddig and Hasenbring, 2017). While PRF questionnaires do not clearly distinguish between these emotions (Lundberg et al., 2011; Kreddig and Hasenbring, 2017), brain research provides evidence for a functional differentiation of fear and anxiety. Both emotions are controlled by the fear circuit (Tovote et al., 2015); however, subcortical regions (e.g., the amygdala) seem to be more involved in fast and defensive fear reactions (short defensive distance to threat) while cortical regions (e.g., the prefrontal cortex) are more likely to be responsible for complex cognitions of anxiety (large defensive distance to threat; McNaughton and Corr, 2004; Qi et al., 2018). Therefore, advances in neuroimaging enable exploring the subcortical/cortical contributions to PRF constructs by examining interrelations between self-reported emotional states and brain response patterns. Specifically, machine learning techniques such as multivariate pattern analysis (MVPA) applied to functional magnetic resonance imaging (fMRI) data make it possible to directly study the predictive relationship between a content-selective cognitive or emotional state (expressed as a label) and corresponding multivoxel fMRI activity patterns (Haynes, 2015; Hebart and Baker, 2017). The label may have discrete (classification) or continuous (regression) values such as questionnaire scores (Formisano et al., 2008). Back-straining activities (i.e., bending and lifting) are the most feared and pain-provoking movements among people with low back pain (LBP), based on ratings of perceived harmfulness or physiologic responses (Leeuw et al., 2007a; Glombiewski et al., 2015; Stevens et al., 2016; Caneiro et al., 2017). As such, bending and lifting, either active or passive (e.g., through pictures) have been frequently used to provoke PRF (Leeuw et al., 2007c; Trost et al., 2009; Barke et al., 2016; Caneiro et al., 2017). Therefore, we provoked PRF by presenting video clips of daily activities including bending and lifting (harmful condition) and harmless activities such as walking (harmless condition) in a sample of 20 patients with nonspecific chronic LBP. We applied a pattern regression analysis in combination with multiple kernel learning (MKL) to assess potential neural predictors of the various PRF constructs based on the weighting of (1) harmful and harmless conditions (condition weights) and (2) pattern information within subcortical and cortical fear-processing regions (region weights). We first contrasted the different PRF questionnaires in terms of their model performance, namely the ability of the model to predict the questionnaire scores based on brain response patterns across fear-processing regions. Second, we compared the different prediction models according to the distributions of their condition and region weights to explore potential neural commonalities or differences of related PRF constructs. If the PRF questionnaires share overlapping PRF constructs, then the region weights should be similarly distributed across fear-processing regions. Conversely, if the contributing brain regions vary across the prediction models, this would provide evidence for nonoverlapping PRF constructs across questionnaires. Ultimately, this approach might help to further understand and dissect the various PRF constructs in chronic LBP.

## Materials and Methods

### Patients

The study was approved by the Ethics Committee Zurich (Switzerland), and all patients provided written informed consent before participation. The study was conducted in accordance with the Declaration of Helsinki. We recruited a total of 20 patients (mean age, 39.35 years; SD, 13.97 years; 7 females; Table 1) with nonspecific chronic LBP, which is considered to be a complex biopsychosocial condition (Deyo and Weinstein, 2001; Maher et al., 2017). Patients were recruited via local chiropractic and physiotherapy centers as well as via on-line advertisements. Inclusion criteria were low back pain of at least 6 months duration and age between 18 and 65 years. Exclusion criteria were a history of psychiatric or neurologic disorders and specific causes for the pain (e.g., infection, tumor, fracture, inflammatory disease) that were ruled out by an experienced clinician.

**Table 1:** Patient characteristics and descriptive statistics of questionnaires

	cLBP patients (*N* = 20, 7 females)			
	Minimum	Maximum	Mean	SD
Age	21	62	39.35	13.97
TSK-17	26	52	36.90	5.59
TSK-13	16	43	27.60	5.96
TSK-11	13	38	23.20	5.71
TSK-11-SF	5	16	9.70	2.69
TSK-11-AA	5	20	11.90	3.35
PASS	13	68	38.15	16.57
PASS-C	1	15	8.70	4.19
PASS-E	3	21	9.85	4.77
PASS-F	2	20	9.45	5.28
PASS-P	0	15	7.35	4.21
FABQ	3	83	35.45	22.53
FABQ-PA	2	21	12.80	5.59
FABQ-W	0	40	15.50	12.12
S-Anxiety	36	53	43.70	4.78
T-Anxiety	31	59	43.00	6.05
PainDETECT current pain	0	8	3.77	2.49
PainDETECT strongest pain	2	10	6.15	2.16
PainDETECT average pain (previous 4 weeks)	1	7	3.75	1.88
Ratings harmful activities	0	10	5.44	2.38
Ratings harmless activities	0	5	1.28	1.32

cLBP = chronic low back pain. Tampa Scale of Kinesiophobia (TSK, SF = somatic focus, AA = activity avoidance); Pain Anxiety Symptoms Scale (PASS, PASSc = cognitive anxiety; PASSe = escape/avoidance; PASSf = fear; PASSp = physiology); Fear Avoidance Beliefs Questionnaire (FABQ, FABQ-PA = physical activity, FABQ-W = work); State-Trait Anxiety Inventory (S-Anxiety, T-Anxiety).

### Self-report measures of pain-related fear

PRF was assessed using several questionnaires:

(1) The Tampa Scale of Kinesiophobia (TSK) questionnaire (Kori et al., 1990; Vlaeyen et al., 1995) was used to assess fear of movement/(re)injury and kinesiophobia. The 17-item German version of the TSK (TSK-17) with satisfactory internal consistency (Cronbach’s α = 0.76–0.84) contains statements focusing on the fear of physical activity rated on a 4-point Likert scale from 1 (strongly disagree) to 4 (strongly agree; Rusu et al., 2014). Due to additional versions of the original 17-item TSK questionnaire, we also calculated the questionnaire scores of the 13- and 11-item TSK versions (TSK-13, TSK-11). The 13- and 11-item versions were previously validated by confirmatory factor analysis and demonstrated acceptable levels of internal consistency (Cronbach’s α = 0.80; Goubert et al., 2004; Tkachuk and Harris, 2012). A two-factor solution of the TSK-11 version provides the best fit in terms of explaining variance across German, Dutch, Swedish, and Canadian samples, and included the subscales “activity-avoidance” (TSK-AA; the belief that that activity may result in injury/reinjury or stronger pain) and “somatic focus” (TSK-SF; the belief in underlying and serious medical problems; Roelofs et al., 2007; Rusu et al., 2014).

(2) The German version of the fear avoidance beliefs questionnaire (FABQ; Waddell et al., 1993; Pfingsten et al., 2000) consists of 16 back pain-specific items related to fear avoidance beliefs rated on a 7-point rating scale (0, completely disagree; 6, completely agree). It includes two distinct and established subscales related to beliefs about about how work (FABQ-W) and physical activity (FABQ-PA) affect LBP, with internal consistencies of α = 0.88 and α = 0.77, respectively (Waddell et al., 1993).

(3) The short version of the pain anxiety symptoms scale (PASS-20) assesses fear and anxiety responses related to pain including cognitive, physiologic, and motor response domains (McCracken and Dhingra, 2002). Items on the PASS-20 are measured on a 6-point Likert scale and relate to four different subscales, including cognitive anxiety (PASS-C), fear (PASS-F), physiology (PASS-P), and escape/avoidance (PASS-E; Roelofs et al., 2004b). The German version of the PASS-20 has an internal consistency of α = 0.90 (Kreddig et al., 2015).

Furthermore, patients were asked to fill out the painDETECT questionnaire, which includes three 11-point numeric rating scales, with 0 being “no pain” and 10 being the “worst imaginable pain” to assess current pain, strongest, and average pain intensity in the previous 4 weeks (Freynhagen et al., 2006). Finally, to investigate potential differences or shared variance between PRF and general anxiety, we used the State-Trait Anxiety Inventory (STAI), the most widely used self-report measure of anxiety, which includes two subscales (Spielberger and Gorsuch, 1983; Julian, 2011): the State Anxiety Scale (S-Anxiety) assesses current levels of anxiety, whereas the Trait Anxiety Scale (T-Anxiety) evaluates more stable aspects of anxiety such as “anxiety proneness” (Julian, 2011). All questionnaires were administered at the fMRI appointment before brain scanning. We tested the scores of the different questionnaires for the assumption of normality of the data using the Shapiro–Wilk test and visually using Q–Q plots implemented in SPSS Statistics (version 23, IBM; Ghasemi and Zahediasl, 2012).

### Scanning protocol and design

Brain imaging was performed on a 3 T whole-body MRI system (Achieva, Philips), equipped with a 32-element receiving head coil and MultiTransmit parallel RF transmission. Each imaging session started with a survey scan, a B1 calibration scan (for MultiTransmit), and a SENSE reference scan. High-resolution anatomic data were obtained with a 3D T1-weighted (T1w) turbo field echo scan consisting of 145 slices in sagittal orientation with the following parameters: field of view (FOV) = 230 × 226 mm^2^; slice thickness = 1.2 mm; acquisition matrix = 208 × 203 (resulting in a voxel resolution of 1.1 × 1.1 × 1.2 mm); TR = 6.8 ms; TE = 3.1 ms; flip angle = 9°; number of signal averages = 1. Functional time series were acquired using whole-brain gradient-echo echoplanar imaging sequences (365 volumes), consisting of 37 slices in the axial direction (anterior commissure–posterior commissure angulation) with the following parameters: FOV = 240 × 240 mm^2^; acquisition matrix = 96 × 96; slice thickness = 2.8 mm (resulting in a voxel resolution of 2.5 × 2.5 × 2.8 mm); interleaved slice acquisition; no slice gap; TR = 2100 ms; TE = 30 ms; SENSE factor = 2.5; flip angle = 80°.

The PRF-provoking stimuli (harmful condition) consisted of video clips with a duration of 4 s recorded from a third-person perspective (Meier et al., 2016). The video clips showed potentially harmful activities (back-straining movements such as bending and lifting) selected from the Photograph Series of Daily Activities (PHODA; Leeuw et al., 2007a). The original PHODA was developed in close collaboration with human movement scientists, physical therapists, and psychologists, and is composed of a fear hierarchy based on ratings of the perceived harmfulness of daily activities in patients with chronic LBP. From the 40 potentially harmful activities included in the short electronic PHODA version (Leeuw et al., 2007a), we chose three scenarios from the top six most harmful activities, namely shoveling soil with a bent back, lifting a flowerpot with slightly bent back, and vacuum cleaning under a coffee table with a bent back. Furthermore, we created video clips of three activities rated as less harmful, such as walking up and down the stairs and walking on even ground (harmless condition). Presentation software (Neurobehavioral Systems) was used to present the video clips in a pseudorandomized order (no more than two identical consecutive trials). The patients were asked to carefully observe the video clips, which were displayed using MR-compatible goggles (Resonance Technology). The three harmful and harmless activities were each presented five times (30 trials total). After the observation of each video clip, the patients were asked to rate the perceived harmfulness of the activity on a visual analog scale (VAS) anchored with the endpoints “not harmful at all” (0) and “extremely harmful” (10). All ratings were performed using an MR-compatible track ball (Current Designs). After the VAS rating, a black screen with a green fixation cross appeared (duration jittered between 6 and 8 s). We have used this experimental protocol successfully for investigations of neural correlates of PRF self-reports in previous fMRI studies based on mass-univariate analyses (Meier et al., 2016, 2017).

### MR data organization and preprocessing

We used an existing fMRI dataset of previously reported studies (Meier et al., 2016, 2017). The fMRI data were organized according to the Brain Imaging Data Structure (RRID:SCR_016124; http://bids.neuroimaging.io/), which provides a consensus on how to organize data obtained in neuroimaging experiments. Preprocessing was performed using FMRIPREP (version 1.0.0-rc2, RRID:SCR_016216; https://github.com/poldracklab/fmriprep), a Nipype based tool (Gorgolewski et al., 2011), which requires minimal user input and provides easily interpretable and comprehensive error and output reporting. This processing pipeline includes state-of-the-art software packages for each step of preprocessing (for a detailed description of the different workflows, see https://fmriprep.readthedocs.io/en/stable/workflows.html). Each T1w volume was skullstripped using antsBrainExtraction.sh version 2.1.0 (using OASIS template). The skullstripped T1w volume was coregistered to the skullstripped ICBM 152 Nonlinear Asymmetrical MNI template version 2009c using nonlinear transformation implemented in ANTs version 2.1.0 (Avants et al., 2008). Functional data were slice time corrected using AFNI (Cox, 1996) and motion corrected using MCFLIRT version 5.0.9 (Jenkinson et al., 2002). This was followed by coregistration to the corresponding T1w volume using boundary-based registration 9 df implemented in FreeSurfer version 6.0.0 (Greve and Fischl, 2009). Motion-correcting transformations, T1w transformation, and MNI template warp were applied in a single step using antsApplyTransformations version 2.1.0 with Lanczos interpolation. Three tissue classes were extracted from T1w images using FSL FAST version 5.0.9 (Zhang et al., 2001). Voxels from CSF and white matter were used to create a mask used to extract physiologic noise regressors using aCompCor (Behzadi et al., 2007). The mask was eroded and limited to subcortical regions to limit overlap with gray matter, and six principal components were estimated. Independent component analysis-based automatic removal of motion artifacts (AROMA) was used to generate aggressive motion-related noise regressors. The AROMA classifier identifies motion components with high accuracy and robustness and is superior to motion artifact detection using 24 motion parameters or spike regression (Pruim et al., 2015). Finally, to preserve high spatial frequency while reducing noise, spatial smoothing with a full-width at half-maximum 4 mm Gaussian kernel was applied. To accelerate data preprocessing, we performed parallel computing using the Docker environment (https://www.docker.com/) and the GC3Pie framework (https://github.com/uzh/gc3pie) on the ScienceCloud supercomputing environment at the University of Zurich (S3IT; https://www.s3it.uzh.ch/).

### MVPA input data

The preprocessed data were subsequently passed onto the Statistical Parametric Mapping software package (SPM12, version 6906; RRID:SCR_007037; http://www.fil.ion.ucl.ac.uk/spm/) for model computation using a general linear model (GLM). For each patient, a design matrix was built with separate regressors for the harmful and harmless activities, respectively (15 harmful and 15 harmless stimuli). The video clips were modeled as boxcar functions (onset = onset of video clip; duration = 4 s) and convolved with the standard canonical hemodynamic response function, as implemented in SPM12. In addition, the following nuisance regressors were implemented in the GLM model for each patient: (1) the six regressors derived from the component-based physiologic noise correction method (aCompCor) and (2) the motion-related regressors generated by AROMA (see MR data organization and preprocessing section). A high-pass filter with a cutoff of 128 s was used to remove low-frequency noise. Finally, for each patient, voxelwise β images for each condition were computed and served as the input images for the MVPA.

### Multivariate pattern analysis

Compared with univariate analyses, MVPA can achieve greater sensitivity and is able to detect subtle and spatially distributed effects (Schrouff et al., 2013; Haynes, 2015). A pattern of activity can represent many more different states than each voxel individually, which leads to an information-based view compared with the activation-based view of univariate analyses (Hebart and Baker, 2017). MVPA was performed using routines implemented in PRoNTo version 2.0 (RRID:SCR_006908; http://www.mlnl.cs.ucl.ac.uk/pronto/; Schrouff et al., 2013). For the readout of multivariate neural information that might serve as a potential score estimator of the different PRF questionnaires, we applied a newly introduced pattern regression approach based on supervised machine learning and testing phases using MKL. In brief, the objective in supervised pattern recognition regression analysis is to learn a function from data that can accurately predict the continuous values (labels; i.e., *f*(*x_i_*) = *y_i_* from a given dataset *D* = {*x_i_*, *y_i_*}, *i* = 1…*N*, where *x_i_* represents pairs of samples or vectors and *y_i_* represents the different labels). Ultimately, the learned function from the learning set is used to predict the labels from new and unseen data (Schrouff et al., 2013). MKL allows accounting for brain anatomy (determined by a brain atlas; see Feature selection) and different modalities (e.g., anatomic/functional data or in the current approach: conditions) during the model estimation by considering each brain region and modality as separate kernels. This approach allows determination of the contribution of each brain region (region weights) and condition (condition weights) to the final decision function of the model in a hierarchical manner by simultaneously learning and combining the different linear kernels that are based on support vector machines (SVMs; Rakotomamonjy et al., 2008; Fernandes et al., 2017; Schrouff et al., 2018). Compared with conventional MVPA methods based on whole-brain voxel weight maps, this procedure provides a straightforward approach to draw inferences on the region level without the need for multiple comparison correction (Schrouff et al., 2018). To account for possible differential contributions of the harmful and harmless conditions to the decision function, we included the individual SPM β images of each condition as separate modalities in the MKL model (condition weights). The kernels were mean centered and normalized (to account for the different sizes of the involved brain regions) using standard routines implemented in PRoNTo. Subsequently, for each questionnaire, we trained a separate MKL regression model with the respective labels (FABQ, TSK-17-, TSK-13, TSK-11, PASS and all subscale scores, and state and trait anxiety). Furthermore, we trained MKL regression models based on the harmfulness ratings collected during the fMRI measurements (mean ratings of the harmful condition and harmless condition, respectively). This resulted in a total of 17 MKL models providing outputs for model evaluation, including model performance, region, and conditions weights. To reduce the risk of overfitting for each model, we applied a nested cross-validation procedure using a “leave-one-subject-out” cross-validation scheme to train the model including optimization of the model hyperparameter “C” (range, [0.1, 1, 10, 100, 1000]). Furthermore, to generate a data-based null distribution of the performance measures [*r* and normalized mean squared error (nMSE); see Model evaluation and interpretation], each model was recomputed 16,000 times with permuted labels (permuted questionnaire score per subject) using parallel computing. Multiple-comparison correction for the model performance (*r* values and nMSE) was based on a false discovery rate (FDR) of 5% (*p*(FDR) < 0.05). As a note, by controlling the expected proportion of false-positives, FDR-controlling procedures provide less stringent control of type I errors compared with other procedures, such as the Bonferroni correction, which control the probability of at least one type I error. In addition, each model representing a potential PRF construct [i.e., a model with a significant (FDR corrected and uncorrected) performance] was trained and tested through an additional cross-validation procedure using each predictive feature set (brain regions that contributed >10%; see Table 5) of the other models (between-model cross-validation; e.g., training and testing of the FABQ labels was repeated using the predictive feature sets of the TSK-11, TSK-13, and T-Anxiety models). A failure of predictive performance in the between-model cross-validation would point toward a dissociation of brain regions contributing to the different models and would therefore be indicative of nonoverlapping PRF constructs.

### Feature selection

To further reduce the risk of overfitting and based on a priori knowledge of brain regions involved in fear processing, we limited the feature space to bilateral fear-related brain regions including the amygdala, hippocampus, thalamus, anterior cingulate, insula, and medial prefrontal, and orbitofrontal cortices (Meier et al., 2014; Tovote et al., 2015; Braem et al., 2017). The respective brain regions were parcellated according to the automated anatomic labeling (AAL; RRID:SCR_003550; http://www.gin.cnrs.fr/en/tools/aal-aal2/; see Table 5 for the different labels; Tzourio-Mazoyer et al., 2002) atlas and projected on the ICBM 152 Nonlinear template (see MR data organization and preprocessing) by means of MATLAB (version R2017b)-based surface-volume registration tools (svreg) implemented in BrainSuite (version 17a; RRID:SCR_006623; http://brainsuite.org/; Shattuck and Leahy, 2002). BrainSuite was also used to generate surfaces of the selected AAL regions for visualization.

### Model evaluation and interpretation

Model performance was assessed by two metrics commonly used to assess the performance of regression models (Ivanescu et al., 2016; Fernandes et al., 2017), as follows: Pearsons’s correlation coefficient (*r*) and the MSE. The correlation coefficient characterizes the linear relationship between true and predicted labels; the MSE is calculated as the average of the squared differences between the true and predicted labels. A significant positive correlation between true and predicted labels would indicate strong decoding performance. Unlike in conventional correlation analysis, however, a negative correlation would indicate poor performance. Furthermore, for each model, we report the nMSE because the different questionnaires are based on different score ranges. To explore possible differential contributions of fear-related brain regions to the prediction models, we report the contribution rank of each brain region (region weight) within each condition (condition weight) provided by the MKL approach (see Table 5). Importantly, the selection of regions by the MKL model might be influenced by small variations in the dataset (because of the leave-one-subject-out cross-validation) and might therefore lead to different subsets of regions being selected across cross-validation steps (folds). Providing a quantification of this variability, the “expected ranking” (ER; see Table 5) characterizes the stability of the region ranking across folds, as follows: The closer the ER to the ranking of the selected fold, the more consistent is the ranking of the respective brain region across folds. On the other hand, if the ER is different from the ranking, this means that the ranking might be variable across folds.

## Results

### Ratings, questionnaire scores, and correlations

Importantly, the comparison of the ratings during fMRI measurements demonstrated that the potentially harmful activities were perceived as being significantly more harmful compared with the harmless activities (paired *t* test: T = 8.22; *p* < 0.001, two-tailed). Descriptive statistics of the different questionnaires as well as the age and sex of the patients are summarized in Table 1. Regarding the questionnaire data, visual inspection (Q-Q plots) and the Shapiro–Wilk test indicated the non-normality of the data (*p* < 0.05) of several questionnaires (FABQ, FABQ-W, TSK-11, FABQ-PA, and T-Anxiety); therefore, the nonparametric Spearman’s rank correlation coefficient was used. Several significant positive correlations between the different PRF questionnaires scores were observed (*p* < 0.05; Table 2). Most of the TSK scales significantly correlated with the PASSs (0.97 < *r*'s > 0.46, *p* < 0.05), whereas the FABQ work scale did not show significant relationships with the TSK and PASSs (*p* > 0.05), except for the PASS-F (*r* = 0.49, *p* < 0.05). Furthermore, only the S-Anxiety scale of the STAI scale demonstrated significant correlations with some, but not all, TSK scales (0.44 > *r*'s < 0.63, *p* < 0.05). Finally, only the PASS-F showed a positive and significant relationship with the mean rating of the harmful condition (*r* = 0.44, *p* < 0.05; Table 2).

**Table 2:** Spearman’s rank correlations between the different pain-related fear questionnaires

** **		**TSK-17**	**TSK-13**	**TSK-11**	**TSK-11_SF**	**TSK-11_AA**	**PASS**	**PASS-C**	**PASS-E**	**PASS-F**	**PASS-P**	**FABQ**	**FABQ-PA**	**FABQ-W**	**S-ANXIETY**	**T-ANXIETY**	**Rating harmful**	**Rating harmless**
**TSK-17**	Corr. coeff.	1.000	**0.834** **	**0.800** **	**0.609** **	**0.759** **	**0.611** **	0.503^*^	**0.614** **	0.556^*^	**0.647** **	0.337	0.494^*^	0.280	0.449^*^	0.299	0.133	0.289
	Sig.		**0.000**	**0.000**	**0.004**	**0.000**	**0.004**	**0.024**	**0.004**	**0.011**	**0.002**	0.146	**0.027**	0.231	**0.047**	0.200	0.577	0.216
**TSK-13**	Corr. coeff.	**0.834** **	1.000	**0.960** **	**0.754** **	**0.789** **	**0.686** **	0.559^*^	**0.777** **	**0.666** **	0.558^*^	0.344	0.442*	0.339	0.451^*^	0.139	0.240	0.168
	Sig.	**0.000**		**0.000**	**0.000**	**0.000**	**0.001**	**0.010**	**0.000**	**0.001**	**0.011**	0.138	**0.050**	0.144	**0.046**	0.558	0.307	0.479
**TSK-11**	Corr. coeff.	**0.800** **	**0.960** **	1.000	**0.793** **	**0.779** **	**0.685** **	0.559^*^	**0.766** **	**0.643** **	**0.565** **	0.360	0.404	0.378	0.470^*^	0.071	0.276	0.091
	Sig.	**0.000**	**0.000**		**0.000**	**0.000**	**0.001**	**0.010**	**0.000**	**0.002**	**0.009**	0.120	0.077	0.101	**0.037**	0.766	0.238	0.703
**TSK-11_SF**	Corr. coeff.	**0.609** **	**0.754** **	**0.793** **	1.000	0.350	0.519^*^	0.462^*^	0.529^*^	0.502^*^	0.502^*^	0.351	0.315	0.411	**0.629** **	0.034	0.044	0.178
	Sig.	**0.004**	**0.000**	**0.000**		0.131	**0.019**	**0.040**	**0.016**	**0.024**	**0.024**	0.130	0.176	0.071	**0.003**	0.886	0.854	0.452
**TSK-11_AA**	Corr. Coeff.	**0.759** **	**0.789** **	**0.779** **	0.350	1.000	0.477^*^	0.268	**0.564** **	0.486^*^	0.421	0.336	0.375	0.280	0.284	0.035	0.270	0.135
	Sig.	**0.000**	**0.000**	**0.000**	0.131		**0.034**	0.254	**0.010**	**0.030**	0.065	0.147	0.103	0.231	0.224	0.883	0.250	0.570
**PASS**	Corr. coeff.	**0.611** **	**0.686** **	**0.685** **	0.519^*^	0.477^*^	1.000	**0.895** **	**0.899** **	**0.886** **	**0.801** **	0.415	0.535^*^	0.400	0.320	0.156	0.317	-0.118
	Sig.	**0.004**	**0.001**	**0.001**	**0.019**	**0.034**		**0.000**	**0.000**	**0.000**	**0.000**	0.069	**0.015**	0.081	0.168	0.510	0.173	0.620
**PASS-C**	Corr. coeff.	0.503^*^	0.559^*^	0.559^*^	0.462^*^	0.268	**0.895** **	1.000	**0.737** **	**0.690** **	**0.707** **	0.329	0.424	0.344	0.227	0.118	0.214	-0.254
	Sig.	**0.024**	**0.010**	**0.010**	**0.040**	0.254	**0.000**		**0.000**	**0.001**	**0.000**	0.157	0.063	0.137	0.336	0.621	0.366	0.280
**PASS-E**	Corr. coeff.	**0.614** **	**0.777** **	**0.766** **	0.529^*^	**0.564** **	**0.899** **	**0.737** **	1.000	**0.918** **	0.544^*^	0.472^*^	**0.592** **	0.419	0.387	0.161	0.330	-0.062
	Sig.	**0.004**	**0.000**	**0.000**	**0.016**	**0.010**	**0.000**	**0.000**		**0.000**	**0.013**	**0.036**	**0.006**	0.066	0.092	0.499	0.156	0.795
**PASS-F**	Corr. coeff.	0.556^*^	**0.666** **	**0.643** **	0.502^*^	0.486^*^	**0.886** **	**0.690** **	**0.918** **	1.000	0.541^*^	**0.577** **	**0.736** **	0.486^*^	0.291	0.188	0.445^*^	0.085
	Sig.	**0.011**	**0.001**	**0.002**	**0.024**	**0.030**	**0.000**	**0.001**	**0.000**		**0.014**	**0.008**	**0.000**	**0.030**	0.213	0.428	**0.049**	0.720
**PASS-P**	Corr. coeff.	**0.647** **	0.558^*^	**0.565** **	0.502^*^	0.421	**0.801** **	**0.707** **	0.544^*^	0.541^*^	1.000	0.261	0.304	0.328	0.289	0.112	0.118	-0.023
	Sig.	**0.002**	**0.011**	**0.009**	**0.024**	0.065	**0.000**	**0.000**	**0.013**	**0.014**		0.267	0.193	0.157	0.216	0.639	0.619	0.925
**FABQ**	Corr. coeff.	0.337	0.344	0.360	0.351	0.336	0.415	0.329	0.472^*^	**0.577** **	0.261	1.000	**0.781** **	**0.951** **	0.314	-0.032	0.195	0.009
	Sig.	0.146	0.138	0.120	0.130	0.147	0.069	0.157	**0.036**	**0.008**	0.267		**0.000**	**0.000**	0.178	0.894	0.410	0.970
**FABQ-PA**	Corr. coeff.	0.494^*^	0.442*	0.404	0.315	0.375	0.535^*^	0.424	**0.592** **	**0.736** **	0.304	**0.781** **	1.000	**0.638** **	0.140	0.009	0.377	0.178
	Sig.	**0.027**	**0.050**	0.077	0.176	0.103	**0.015**	0.063	**0.006**	**0.000**	0.193	**0.000**		**0.002**	0.557	0.969	0.101	0.452
**FABQ-W**	Corr. coeff.	0.280	0.339	0.378	0.411	0.280	0.400	0.344	0.419	0.486^*^	0.328	**0.951** **	**0.638** **	1.000	0.291	-0.069	0.185	-0.040
	Sig.	0.231	0.144	0.101	0.071	0.231	0.081	0.137	0.066	**0.030**	0.157	**0.000**	**0.002**		0.213	0.772	0.435	0.867
**S-ANXIETY**	Corr. coeff.	0.449^*^	0.451^*^	0.470^*^	**0.629** **	0.284	0.320	0.227	0.387	0.291	0.289	0.314	0.140	0.291	1.000	0.128	-0.198	0.090
	Sig.	**0.047**	**0.046**	**0.037**	**0.003**	0.224	0.168	0.336	0.092	0.213	0.216	0.178	0.557	0.213		0.592	0.402	0.707
**T-ANXIETY**	Corr. coeff.	0.299	0.139	0.071	0.034	0.035	0.156	0.118	0.161	0.188	0.112	-0.032	0.009	-0.069	0.128	1.000	0.185	0.378
	Sig.	0.200	0.558	0.766	0.886	0.883	0.510	0.621	0.499	0.428	0.639	0.894	0.969	0.772	0.592		0.435	0.100
**Rating harmful**	Corr. coeff.	0.133	0.240	0.276	0.044	0.270	0.317	0.214	0.330	0.445^*^	0.118	0.195	0.377	0.185	-0.198	0.185	1.000	0.289
	Sig.	0.577	0.307	0.238	0.854	0.250	0.173	0.366	0.156	**0.049**	0.619	0.410	0.101	0.435	0.402	0.435		0.217
**Rating harmless**	Corr. coeff.	0.289	0.168	0.091	0.178	0.135	-0.118	-0.254	-0.062	0.085	-0.023	0.009	0.178	-0.040	0.090	0.378	0.289	1.000
	Sig.	0.216	0.479	0.703	0.452	0.570	0.620	0.280	0.795	0.720	0.925	0.970	0.452	0.867	0.707	0.100	0.217	

Corr. coeff., Correlation coefficient; Sig., significance.

**p < 0.005 (bold), *p < 0.05 (bold). Bold indicates significance.

### Model performance

**Figure 1.**
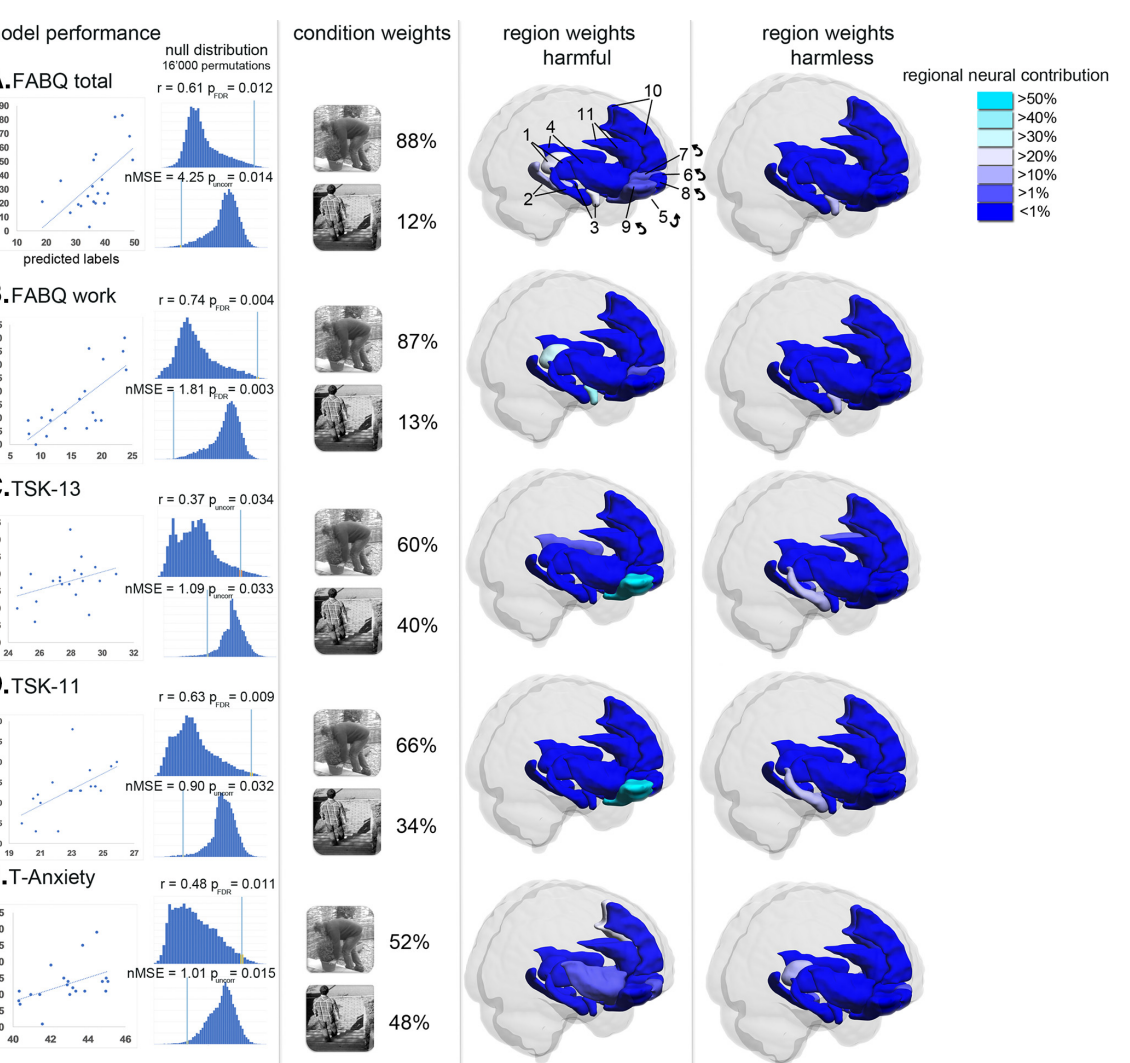
The model performance (*r*, MSE) characterizes the strength of the relationship between true and predicted labels. Condition and region weights show the predictive contribution of the two different conditions (harmful, harmless) and fear-related brain regions (parcellated according to the AAL atlas; L, left; R, right) to the final decision function of each MKL model (questionnaires ***A–E*** with model performance; *p* < 0.05, FDR corrected and uncorrected). Brain regions (feature set) were identified as follows: thalamus (1); hippocampus (2); amygdala (3); insula (4); mOFC: rectus (5), Frontal_Sup_Orb (6), Frontal_Med_Orb (7); lOFC: Frontal_Mid_Orb (8), Frontal_Inf_Orb (9), mPFC: Frontal_Sup_Medial (10); anterior cingulate cortex: Cingulum_Ant (11). ← indicates the not visible contralateral homolog.

The MKL models with significant performance results [*p* < 0.05, FDR-corrected (FDR) and uncorrected (uncorr)] characterized by the Pearsons’s correlation coefficient (*r*) and the nMSE are depicted in Figure 1*A–E* (Table 3, for review). The FABQ model demonstrated a significant decoding performance characterized by a positive correlation between true and predicted labels (*r* = 0.61, *p*(FDR) = 0.012, nMSE = 4.25, *p*(uncorr) = 0.014). Interestingly, the FABQ-W model showed strong predictive power (*r* = 0.74, *p*(FDR) = 0.004, nMSE = 1.81, *p*(FDR) = 0.003), whereas the FABQ-PA scale was not decodable from fear-related brain response patterns (*r* = 0.03, *p*(uncorr) = 0.162, nMSE = 1.68, *p*(uncorr) = 0.161). Among the TSK scales, only the TSK-13 (*r* = 0.37, *p*(uncorr) = 0.034, nMSE = 1.09, *p*(uncorr) = 0.033) and the TSK-11 (*r* = 0.63, *p*(FDR) = 0.009, nMSE = 0.90, *p*(uncorr) = 0.032) models demonstrated a significant decoding performance. The TSK-17 model (*r* = 0.19, *p*(uncorr) = 0.09, nMSE = 1.10, *p*(uncorr) = 0.091) and the TSK-11 subscale models did not show a significant decoding performance (TSK11-SF: (*r* = −0.73, *p*(uncorr) = 0.832, nMSE = 0.86, *p*(uncorr) = 0.773; TSK-11-AA: *r* = −0.63, *p*(uncorr) = 0.908, nMSE = 0.88, *p*(uncorr) = 0.879). In addition, none of the PASSs were decodable from fear-related brain response patterns (PASS: *r* = 0.18, *p*(uncorr) = 0.119, nMSE = 4.63, *p*(uncorr) = 0.115/PASS-C: *r* = −0.44, *p*(uncorr) = 0.515, nMSE = 1.64, *p*(uncorr) = 0.513/PASS-E: *r* = −0.32, *p*(uncorr) = 0.339, nMSE = 1.38, *p*(uncorr) = 0.331, PASS-F: *r* = −0.15, *p*(uncorr) = 0.259, nMSE = 1.70, *p*(uncorr) = 0.251/PASS-P: *r* = −0.51, *p*(uncorr) = 0.518, nMSE = 1.36, *p*(uncorr) = 0.512). Furthermore, the T-Anxiety model demonstrated a moderate decoding performance (*r* = 0.48, *p*(FDR) = 0.011, nMSE = 1.01, *p*(uncorr) = 0.015), whereas the S-Anxiety model was not significant (*r* = −0.46, *p*(uncorr) = 0.481, nMSE = 1.51, *p*(uncorr) = 0.475). The ratings of perceived harmfulness during fMRI measurements were not decodable from fear-related brain response patterns (rating harmful: *r* = −0.01, *p*(uncorr) = 0.247, nMSE = 0.64, *p*(uncorr) = 0.242; Rating harmless: *r* = −0.72, *p*(uncorr) = 0.481, nMSE = 0.38, *p*(uncorr) = 0.441). Finally, the between-model cross-validation (see Multivariate pattern analysis) did not result in significant performance results (*p*(uncorr) values >0.11) between different feature sets (e.g., FABQ labels were not predictable using the TSK-11 feature set; Table 4).

**Table 3:** Model performances of the different MKL models (characterized by *r* and nMSE)

MKL model	*r*	*p* value	nMSE	*p* value
FABQ total	**0.61**	0.012*	**4.25**	**0.014**
FABQ-W	**0.74**	0.004*	**1.81**	0.003*
FABQ-PA	0.03	0.162	1.68	0.161
TSK-17	0.19	0.098	1.10	0.091
TSK-13	**0.37**	**0.034**	**1.09**	**0.033**
TSK-11	**0.63**	0.009*	**0.90**	**0.032**
TSK-11-SF	−0.73	0.832	0.86	0.773
TSK-11-AA	−0.63	0.908	0.88	0.879
PASS	0.18	0.119	4.63	0.115
PASS-C	−0.44	0.515	1.64	0.513
PASS-E	−0.32	0.339	1.38	0.331
PASS-F	−0.15	0.259	1.70	0.251
PASS-P	−0.51	0.518	1.36	0.512
T-Anxiety	**0.48**	0.011*	**1.01**	**0.015**
S-Anxiety	−0.46	0.481	1.51	0.475
Ratings harmful activities	−0.01	0.247	0.64	0.242
Ratings harmless activities	−0.72	0.481	0.38	0.441

Bold: *p* < 0.05, * *p* < 0.05, corrected for multiple comparisons (FDR, 5%).

**Table 4:** Model performances of the between-model cross-validation (characterized by *r* and nMSE)

**Feature set**	**FABQ total labels**	**FABQ-W labels**	**TSK-11 labels**	**TSK-13 labels**	**T-Anxiety labels**
**FABQ total**		*r* = 0.72, *p* = 0.001*, nMSE = 3.29, *p* = 0.001*	*r* = −0.42, *p* = 0.68 nMSE = 1.30, *p* = 0.69	*r* = −0.65, *p* = 0.91 nMSE = 1.48, *p* = 0.86	*r* = −0.32, *p* = 0.42 nMSE = 1.56, *p* = 0.66
**FABQ-W**	*r* = 0.8, *p* = 0.001*, nMSE = 1.63, *p* = 0.001*		*r* = −0.40, *p* = 0.59, nMSE = 1.26, *p* = 0.47	*r* = −0.06, *p* = 0.25 nMSE = 4.08, *p* = 0.24	*r* = −0.32, *p* = 0.42 nMSE = 1.56, *p* = 0.66
**TSK-11**	*r* = 0.17, *p* = 0.12, nMSE = 6.27, *p* = 0.13	*r* = −0.06, *p* = 0.26, nMSE = 4.08, *p* = 0.26		*r* = 0.83, *p* = 0.001* nMSE = 0.57, *p* = 0.002*	*r* = −0.39, *p* = 0.57 nMSE = 1.74, *p* = 0.96
**TSK-13**	*r* = 0.04, *p* = 0.18, nMSE = 7.16, *p* = 0.32	*r* = −0.06, *p* = 0.31, nMSE = 4.08, *p* = 0.27	*r* = 0.83, *p* = 0.001* nMSE = 0.57, *p* = 0.001*		*r* = −0.37, *p* = 0.47 nMSE = 1.78, *p* = 0.98
**T-Anxiety**	*r* = 0.23, *p* = 0.11, nMSE = 6.01, *p* = 0.11	*r* = 0.2, *p* = 0.21, nMSE = 3.43, *p* = 0.25	*r* = −0.25, *p* = 0.41, nMSE = 1.35, *p* = 0.81	*r* = −0.24, *p* = 0.41, nMSE = 1.45, *p* = 0.83	

Bold: *p* < 0.05, * *p* < 0.05, corrected for multiple comparisons (FDR of 5%).

### Condition and region weights

The condition and region weights of models with predictive performance (*p* < 0.05, FDR-corrected and uncorrected; see Model performance) are illustrated in Figure 1*A–E* and are described in detail in Table 5 (sections A–E). The decoding performances of the FABQ models (FABQ and FABQ-W) were driven by a major contribution of the harmful condition (88% and 87%, respectively). Within this condition, the left thalamus (rank 1), the right amygdala (rank 2) and the left hippocampus (rank 3) contributed >69% of the total region weights in the FABQ model (Table 5, section A, Fig. 1*A*). Similarly, the right amygdala (rank 1) and the left thalamus (rank 2) carried the most predictive neural information with 79.62% of the total region weights in the FABQ-W model (Table 5, section B, Fig. 1*B*). In both FABQ models, the right amygdala also demonstrated an association with the harmless condition, although it was of minor relevance (∼11%). In comparison, the TSK models demonstrated a moderate contribution of the harmful condition (TSK-13, 60%; TSK-11, 66%). Both predictive model performances of the TSK were driven by a major contribution of the right lateral orbitofrontal cortex (lOFC; TSK-13, 52.7%; TSK-11, 60.49%; Table 5, sections C and D, Fig. 1*C*,*D*). Furthermore, the left medial orbitofrontal cortex (mOFC) and the right hippocampus carried predictive information within the harmless condition in both TSK models (TSK-13: left gyrus rectus, 19.51%; right hippocampus, 14.03%; TSK-11: left gyrus rectus, 21.29%; right hippocampus, 10.41%). With almost equal contributions of the harmful (52%) and harmless conditions (48%), the prediction of the T-Anxiety scores was mainly driven by neural contributions of the left medial prefrontal cortex (mPFC) and mOFC (accounting for 44% of the total region weights in the harmful condition) and the left thalamus (together with the mOFC accounting for 44% of the total region weights in the harmless condition; Table 5, section E, Fig. 1*E*).

**Table 5:** Condition and region weights showing the contribution of the two different conditions and fear-related brain regions to the final decision function of each MKL model (questionnaires A–E with model performance, *p* < 0.05, FDR corrected and uncorrected; see Fig. 1**) in hierarchical order**

	**Harmful activities**	**Condition weight**			**Harmless activities**	**Condition weight**		
**Rank**	**Brain regionAAL label**	**Region size (vox)**	**Region weight (%)**	**ER**	**Brain regionAAL label**	**Region size (vox)**	**Region weight (%)**	**ER**
**A. FABQ total^a^**								
1	Thalamus_L*	519	27.25	1.8	Amygdala_R*	96	11.06	0.95
2	Amygdala_R*	96	24.69	1.6	Hippocampus_R	424	0.61	14.70
3	Hippocampus_L*	400	17.29	2.6	Amygdala_L	97	0.43	18.10
4	Frontal_Med_Orb_R	413	9.56	4.0	Frontal_Inf_Orb_L	714	0.19	5.15
5	Frontal_Inf_Orb_R	744	6.39	6.1	Frontal_Sup_Orb_L	451	0.00	2.35
6	Frontal_Med_Orb_L	324	2.17	7.5	Frontal_Sup_Orb_R	469	0.00	3.30
7	Hippocampus_R	424	0.31	8.2	Frontal_Mid_Orb_L	408	0.00	4.25
8	Frontal_Sup_Orb_L	451	0.00	6.1	Frontal_Mid_Orb_R	444	0.00	5.20
9	Frontal_Sup_Orb_R	469	0.00	7.0	Frontal_Inf_Orb_R	744	0.00	6.90
10	Frontal_Mid_Orb_L	408	0.00	8.0	Frontal_Sup_Medial_L	1417	0.00	7.85
11	Frontal_Mid_Orb_R	444	0.00	8.9	Frontal_Sup_Medial_R	1006	0.00	8.80
12	Frontal_Inf_Orb_L	714	0.00	9.9	Frontal_Med_Orb_L	324	0.00	9.75
13	Frontal_Sup_Medial_L	1417	0.00	11.1	Frontal_Med_Orb_R	413	0.00	10.70
14	Frontal_Sup_Medial_R	1006	0.00	12.1	Rectus_L	381	0.00	11.65
15	Rectus_L	381	0.00	13.3	Rectus_R	352	0.00	12.60
16	Rectus_R	352	0.00	14.3	Insula_L	887	0.00	13.55
17	Insula_L	887	0.00	15.2	Insula_R	821	0.00	14.50
18	Insula_R	821	0.00	16.2	Cingulum_Ant_L	599	0.00	15.45
19	Cingulum_Ant_L	599	0.00	17.1	Cingulum_Ant_R	639	0.00	16.40
20	Cingulum_Ant_R	639	0.00	18.1	Hippocampus_L	400	0.00	17.35
21	Amygdala_L	97	0.00	19.9	Thalamus_L	519	0.00	19.95
22	Thalamus_R	478	0.00	20.9	Thalamus_R	478	0.00	20.90
**B. FABQ-W^b^**								
1	Amygdala_R*	96	40.20	1.40	Amygdala_R*	96	11.82	1.05
2	Thalamus_L*	519	39.42	1.45	Hippocampus_R	424	0.64	16.21
3	Frontal_Med_Orb_L	324	4.17	4.90	Frontal_Med_Orb_L	324	0.22	7.57
4	Frontal_Med_Orb_R	413	2.42	7.25	Cingulum_Ant_R	639	0.16	14.52
5	Hippocampus_L	400	0.52	16.55	Frontal_Sup_Orb_L	451	0.00	2.36
6	Cingulum_Ant_R	639	0.24	16.50	Frontal_Sup_Orb_R	469	0.00	3.31
7	Thalamus_R	478	0.13	20.00	Frontal_Mid_Orb_L	408	0.00	4.26
8	Frontal_Sup_Orb_L	451	0.00	4.30	Frontal_Mid_Orb_R	444	0.00	5.21
9	Frontal_Sup_Orb_R	469	0.00	5.25	Frontal_Inf_Orb_L	714	0.00	6.15
10	Frontal_Mid_Orb_L	408	0.00	6.20	Frontal_Inf_Orb_R	744	0.00	7.10
11	Frontal_Mid_Orb_R	444	0.00	7.15	Frontal_Sup_Medial_L	1417	0.00	8.05
12	Frontal_Inf_Orb_L	714	0.00	8.10	Frontal_Sup_Medial_R	1006	0.00	9.00
13	Frontal_Inf_Orb_R	744	0.00	9.05	Frontal_Med_Orb_R	413	0.00	10.63
14	Frontal_Sup_Medial_L	1417	0.00	10.00	Rectus_L	381	0.00	11.57
15	Frontal_Sup_Medial_R	1006	0.00	10.95	Rectus_R	352	0.00	12.52
16	Rectus_L	381	0.00	12.55	Insula_L	887	0.00	13.47
17	Rectus_R	352	0.00	13.50	Insula_R	821	0.00	14.42
18	Insula_L	887	0.00	14.45	Cingulum_Ant_L	599	0.00	15.36
19	Insula_R	821	0.00	15.40	Hippocampus_L	400	0.00	17.15
20	Cingulum_Ant_L	599	0.00	16.35	Amygdala_L	97	0.00	18.94
21	Hippocampus_R	424	0.00	19.05	Thalamus_L	519	0.00	19.89
22	Amygdala_L	97	0.00	20.00	Thalamus_R	478	0.00	20.84
**C. TSK-13^c^**								
1	Frontal_Inf_Orb_R*	744	52.70	1.55	Rectus_L*	381	19.51	2.00
2	Rectus_L	381	2.37	6.00	Hippocampus_R*	424	14.03	1.80
3	Insula_L	887	1.33	8.90	Amygdala_L	97	2.34	16.40
4	Hippocampus_L	400	0.67	14.05	Cingulum_Ant_L	599	1.09	13.95
5	Insula_R	821	0.62	11.55	Rectus_R	352	0.59	11.65
6	Amygdala_R	96	0.33	17.10	Frontal_Sup_Orb_R	469	0.44	4.50
7	Frontal_Mid_Orb_R	444	0.12	5.60	Hippocampus_L	400	0.41	15.85
8	Frontal_Med_Orb_R	413	0.12	10.35	Thalamus_R	478	0.14	19.55
9	Hippocampus_R	424	0.12	16.50	Frontal_Med_Orb_R	413	0.12	11.25
10	Frontal_Med_Orb_L	324	0.12	9.60	Amygdala_R	96	0.12	18.40
11	Frontal_Inf_Orb_L	714	0.12	6.95	Frontal_Inf_Orb_L	714	0.12	6.95
12	Thalamus_R	478	0.11	20.25	Frontal_Inf_Orb_R	744	0.12	7.90
13	Frontal_Sup_Medial_L	1417	0.11	8.05	Frontal_Sup_Orb_L	451	0.12	3.50
14	Rectus_R	352	0.11	12.15	Frontal_Sup_Medial_L	1417	0.12	8.90
15	Amygdala_L	97	0.11	17.90	Cingulum_Ant_R	639	0.12	16.10
16	Frontal_Sup_Medial_R	1006	0.11	9.20	Frontal_Mid_Orb_R	444	0.12	6.30
17	Frontal_Mid_Orb_L	408	0.11	5.650	Thalamus_L	519	0.11	19.75

18	Thalamus_L	519	0.11	19.80	Frontal_Med_Orb_L	324	0.11	11.00
19	Cingulum_Ant_R	639	0.11	15.50	Insula_R	821	0.11	14.65
20	Frontal_Sup_Orb_R	469	0.11	4.90	Insula_L	887	0.11	13.80
21	Cingulum_Ant_L	599	0.11	14.70	Frontal_Sup_Medial_R	1006	0.11	10.30
22	Frontal_Sup_Orb_L	451	0.00	4.10	Frontal_Mid_Orb_L	408	0.11	5.85
**D. TSK-11^d^**								
1	Frontal_Inf_Orb_R*	744	60.49	1.05	Rectus_L*	381	21.29	1.60
2	Insula_L	887	0.90	11.15	Hippocampus_R*	424	10.41	1.90
3	Amygdala_R	96	0.65	17.20	Thalamus_L	97	0.41	17.60
4	Hippocampus_L	400	0.61	14.95	Amygdala_L	599	0.12	17.25
5	Amygdala_L	97	0.56	17.00	Cingulum_Ant_L	352	0.12	14.65
6	Insula_R	821	0.46	12.55	Thalamus_R	469	0.11	20.00
7	Frontal_Med_Orb_R	413	0.34	9.35	Frontal_Mid_Orb_R	400	0.11	5.75
8	Frontal_Mid_Orb_R	444	0.12	4.90	Cingulum_Ant_R	478	0.11	15.70
9	Frontal_Med_Orb_L	324	0.12	8.65	Frontal_Sup_Medial_L	413	0.11	8.55
10	Hippocampus_R	424	0.11	16.55	Amygdala_R	96	0.11	18.50
11	Rectus_L	381	0.11	10.70	Hippocampus_L	714	0.11	16.75
12	Frontal_Inf_Orb_L	714	0.11	6.30	Frontal_Med_Orb_R	744	0.11	11.45
13	Thalamus_R	478	0.11	20.40	Frontal_Inf_Orb_L	451	0.11	7.00
14	Rectus_R	352	0.11	11.75	Frontal_Sup_Orb_L	1417	0.11	3.45
15	Frontal_Sup_Medial_R	1006	0.11	8.30	Frontal_Inf_Orb_R	639	0.11	8.05
16	Cingulum_Ant_R	639	0.11	15.35	Frontal_Sup_Medial_R	444	0.11	9.90
17	Frontal_Sup_Medial_L	1417	0.11	7.50	Rectus_R	519	0.11	12.65
18	Cingulum_Ant_L	599	0.11	14.55	Insula_R	324	0.11	14.50
19	Frontal_Mid_Orb_L	408	0.11	4.90	Frontal_Sup_Orb_R	821	0.11	4.65
20	Thalamus_L	519	0.11	19.90	Frontal_Mid_Orb_L	887	0.10	5.60
21	Frontal_Sup_Orb_R	469	0.10	4.10	Insula_L	1006	0.10	13.75
22	Frontal_Sup_Orb_L	451	0.00	3.25	Frontal_Med_Orb_L	408	0.10	11.10
**E. T-Anxiety^e^**								
1	Frontal_Sup_Medial_L*	1417	20.20	1.95	Frontal_Med_Orb_L*	324	20.86	1.05
2	Frontal_Med_Orb_L*	324	13.82	1.90	Thalamus_L*	519	13.29	3.75
3	Rectus_L	381	9.97	3.25	Frontal_Sup_Orb_R	469	9.87	2.85
4	Frontal_Mid_Orb_R	444	3.48	7.30	Amygdala_L	97	2.06	10.15
5	Insula_R	821	2.85	6.90	Amygdala_R	96	0.44	18.95
6	Rectus_R	352	0.98	10.40	Frontal_Sup_Medial_L	1417	0.40	9.15
7	Cingulum_Ant_R	639	0.87	14.50	Frontal_Mid_Orb_R	444	0.23	6.30
8	Amygdala_L	97	0.25	17.35	Cingulum_Ant_R	639	0.06	15.95
9	Frontal_Inf_Orb_R	744	0.22	9.00	Hippocampus_L	400	0.00	16.95
10	Amygdala_R	96	0.07	19.00	Frontal_Sup_Orb_L	451	0.00	4.35
11	Frontal_Sup_Orb_L	451	0.00	4.70	Frontal_Mid_Orb_L	408	0.00	5.35
12	Frontal_Sup_Orb_R	469	0.00	5.65	Frontal_Inf_Orb_L	714	0.00	7.25
13	Frontal_Mid_Orb_L	408	0.00	6.60	Frontal_Inf_Orb_R	744	0.00	8.20
14	Frontal_Inf_Orb_L	714	0.00	8.45	Frontal_Sup_Medial_R	1006	0.00	10.10
15	Frontal_Sup_Medial_R	1006	0.00	10.40	Frontal_Med_Orb_R	413	0.00	11.05
16	Frontal_Med_Orb_R	413	0.00	11.35	Rectus_L	381	0.00	12.00
17	Insula_L	887	0.00	13.10	Rectus_R	352	0.00	12.95
18	Cingulum_Ant_L	599	0.00	14.35	Insula_L	887	0.00	13.90
19	Hippocampus_L	400	0.00	16.20	Insula_R	821	0.00	14.85
20	Hippocampus_R	424	0.00	17.15	Cingulum_Ant_L	599	0.00	15.80
21	Thalamus_L	519	0.00	19.95	Hippocampus_R	424	0.00	18.55
22	Thalamus_R	478	0.00	20.90	Thalamus_R	478	0.00	20.90

The brain regions (L, left; R, right) were parcellated according to the AAL atlas: Thalamus, amygdala, hippocampus, medial orbitofrontal regions (mOFC: Rectus, Frontal_Sup_Orb = superior frontal gyrus, orbital part, Frontal_Med_Orb = medial orbitofrontal cortex), lateral orbitofrontal regions (lOFC: Frontal_Mid_Orb = middle frontal gyrus orbital part, Frontal_Inf_Orb = inferior frontal gyrus pars orbitalis), medial prefrontal regions (mPFC: Frontal_Sup_Medial = medial frontal gyrus, Cingulum_Ant = anterior cingulate cortex). ER = expected ranking.

*^a^*Condition weight: harmful activities, 88%; harmless activities, 12%.

*^b^*Condition weight: harmful activities, 87%; harmless activities, 13%.

*^c^*Condition weight: harmful activities, 60%; harmless activities, 40%.

*^d^*Condition weight: harmful activities, 66%; harmless activities, 34%.

*^e^*Condition weight: harmful activities, 52%; harmless activities, 48%.

*Brain regions included in the feature set for between model cross-validation (see Table 4 for results).

## Discussion

Evidence from cross-sectional and longitudinal behavioral studies demonstrates a strong association between PRF and disability in chronic pain (Leeuw et al., 2007b; Wertli et al., 2014b; Esteve et al., 2017). However, the different PRF constructs such as “fear of movement/(re)injury/kinesiophobia,” “fear avoidance beliefs,” or “pain anxiety” are often used interchangeably in the literature (Lundberg et al., 2011), and it is unclear whether they share a common PRF construct reflected by similar neural sources. The subcortical/cortical neural basis of fear and anxiety that controls cognition and regulates appropriate behavior dependent on threat characteristics is well described (Gray and MacNaughton, 2000; LeDoux, 2000; McNaughton and Corr, 2004; Panksepp, 2011; Shackman et al., 2011; Qi et al., 2018). Although both emotions are linked to similar neuromodulatory systems of the fear circuit (Tovote et al., 2015), anxiety is less well understood and more complex than fear. Current research suggests a functional differentiation characterized by subcortical regions processing fast fear responses to an imminent threat (defensive responses) and cortical systems processing complex cognitions related to fear and anxiety where the threat is distal in space or time (LeDoux and Pine, 2016; Qi et al., 2018).

The current MVPA approach using MKL demonstrated the feasibility to neuronally dissect the proposed constructs of PRF self-reports based on their subcortical/cortical predictors during PRF-related brain activity. The results revealed that while the variability across individuals of some questionnaires, specifically the FABQ and FABQ-W, TSK-13, TSK-11, and T-Anxiety scales, was predictable from response patterns in fear-related, dissociable neural sources on subcortical and cortical levels, this was not the case for the FABQ-PA, the TSK-11 subscales (TSK-11-AA and TSK-SF), the PASSs, and the S-Anxiety scale. Furthermore, the on-line ratings of perceived harmfulness were not decodable from fear-related brain response patterns.

### FABQ and TSK

The FABQ and FABQ-W scales demonstrated the best model performances among the investigated PRF questionnaires, which were characterized by a strong contribution of neural information in the harmful condition (condition weights, 88% and 87%, respectively). Interestingly, the FABQ-PA scale did not show a predictive association with fear-related brain response patterns. The better model performance of the FABQ-W based on fear-related brain activity patterns is in line with the emerging evidence that the FABQ-W is a better predictor of treatment outcome in chronic LBP compared with the FABQ-PA, although this might be dependent on the patient population (Waddell et al., 1993; George et al., 2005, 2008; Wertli et al., 2014a). In support of this, the FABQ-W scale qualified for a clinical prediction rule regarding improvement after spinal manipulation, whereas the FABQ-PA scale did not (Flynn et al., 2002; Dougherty et al., 2014).

With respect to the region weights, the FABQ models were mainly driven by subcortical neural contributions involving the thalamus, hippocampus, and the amygdala, while frontal brain regions played a minor role. The thalamus and, particularly, its midline structures have been considered to be a nonspecific arousing system (van der Werf et al., 2002). However, it has been shown recently that parts of dorsal midline thalamic structures are necessary for fear memory processing by directly targeting the hippocampus, which plays an important role for context-dependent emotional memory (Penzo et al., 2015; Lara-Vásquez et al., 2016; Zheng et al., 2017). Furthermore, the amygdala has long been considered a “fear center” (Darwin, 1873; Panksepp, 1998). However, the heterogeneous structure consisting of several nuclei is not essential for the experience of fear, which has been demonstrated in patients with amygdala lesions (Anderson and Phelps, 2002; Feinstein et al., 2013; LeDoux and Pine, 2016). Instead, the amygdala has been shown to be more strongly implicated in behavioral and physiologic responses to threats (i.e., defensive processes); its relation to complex cognitions like fear and anxiety is controversial (Panksepp, 2011; LeDoux and Pine, 2016; Fanselow and Pennington, 2017). A recent opinion article (LeDoux and Hofmann, 2018) suggested that subjective feelings of fear and anxiety do not initially arise from subcortical activity of the fear circuit centered around the amygdala. Thus, amygdala activity and mediated physiologic responses of fear and anxiety might be, at its best, only a correlate of subjective feelings of fear and anxiety (LeDoux and Hofmann, 2018). Nevertheless, the results presented here indicate a strong predictive association between subjective reports of PRF, assessed by the FABQ scales, and amygdala activity patterns.

Among the TSK scales, the TSK-13 and the TSK-11 demonstrated a predictive association with fear-related brain response patterns, albeit with less contribution of the harmful condition compared with the FABQ scales (TSK-13, 60%; TSK-11, 66%). The TSK-11 version showed a stronger relationship between true and predicted labels compared with the TSK-13 version (*r* = 0.60, nMSE = 0.90, *p* < 0.05). This result might reflect the progress of previous research regarding the psychometric properties of the different TSK versions. Compared with the 17-item version, the 13-item version has better psychometric properties without the four inversely phrased items (Roelofs et al., 2004a; Neblett et al., 2016), and the 11-item version has been recommended for future research and clinical settings (for a chronological summary, see Tkachuk and Harris, 2012). Interestingly, no predictive association could be “learned” by MKL using the TSK-11 subscale labels (TSK-11-SF and TSK-11-AA scores). Although these two lower-order factors (activity avoidance and somatic focus) are reflective of the higher-order construct “fear of movement and (re)injury/kinesiophobia,” the nonsignificant result might indicate that they are associated with inconsistent neural patterns across individuals.

Regarding the region weights of the TSK models, the right lOFC provided the most predictive information for the two TSK scales (TSK-13, 52%; TSK-11, 60%). In agreement with the phobia-related construct (kinesiophobia), dysfunction of the OFC has been shown to be implicated in the processing of phobia-related stimuli in disorders such as social anxiety disorder (Dilger et al., 2003). Specifically, lOFC activity was reduced when phobogenic trials were contrasted with fear-relevant trials (Aue et al., 2015). Furthermore, a hyperactive lOFC has been shown to be linked to anxiety-laden cognitions (Hahn et al., 2011). Interestingly, the higher cortical contributions of the TSK models were clearly dissociable from the largely subcortical contributions involving the amygdala, hippocampus, and thalamus that predicted the FABQ scores.

To conclude, the FABQ scales demonstrated high PRF sensitivity (harmful condition weights > 87%) and were linked to subcortical predictors that have been associated with fear responses to an imminent threat and defensive behavior (McNaughton and Corr, 2004; LeDoux and Pine, 2016). In contrast, the TSK scales appeared to capture emotional states largely associated with cortical fear processing that might be related to cognitive aspects of PRF. In support of this, the observed higher harmless condition weights of the TSK compared with the FABQ models might indicate that the TSK scales are associated with more diffuse anxiety-related cognitions.

### PASS

Surprisingly, the PASS failed to demonstrate a predictive association with fear-related brain response patterns. There may be several explanations. First, although the FABQ and the TSK have been specifically developed for patients with musculoskeletal pain, the PASS is suitable for various pain phenotypes (Crombez et al., 1999). Second, the PASS has been shown to be more strongly associated with negative affect and less predictive of pain disability and behavioral performance (Crombez et al., 1999). Third, all PASS subscales demonstrated significant multicollinearity in our sample, suggesting nonindependence between the different subscales. All these aspects may have led to less sensitivity of fear-related neural patterns to the PASS and its subscales in the current study.

The superiority of the FABQ scale (driven by the FABQ-W) in decoding performance compared with the TSK and PASSs might also be influenced by the back-specific items of the FABQ in conjunction with the nature of the PRF-provoking stimuli (back-straining movements). The items of the FABQ were specifically related to the back, while the TSK and PASS can be used with various musculoskeletal pain diagnoses such as work-related upper extremity disorders, chronic LBP, fibromyalgia, and osteoarthritis (Roelofs et al., 2007). Nevertheless, the FABQ has also been adapted to shoulder pain, where it demonstrated better factor structure and a stronger association with disability compared with the TSK-11 (Mintken et al., 2010).

### State and trait anxiety

Beside PRF, anxiety, and depression significantly mediate the relationship between pain and disability (Marshall et al., 2017). However, fear responses specifically related to a patient’s pain and/or potentially painful movements might be more relevant for explaining disability in chronic LBP than general trait anxiety responses (McCracken et al., 1996). The current results are in line with this notion. First, most of the PRF measures did not show a significant relationship with state or trait anxiety. Second, state anxiety was not decodable from fear-related brain responses to potentially harmful activities in chronic pain patients. Interestingly, with respect to the trait anxiety model (T-Anxiety; Fig. 1*E*), the harmful and the harmless conditions carried almost equal predictive neural information (52% vs 48%). This suggests that the trait anxiety measure is associated with neural content irrespective of the harmfulness of a stimulus, provoked by, for example, enhanced attention to visual information processed in fear-related brain regions; Berggren et al., 2015). It might further indicate that the T-Anxiety scale captures neural responses that are associated with a more generalized fear response.

Regarding the region weights, predictive information was predominantly provided by brain regions that were less involved in the prediction of the other PRF measures, namely parts of the mPFC and mOFC (Table 5, section E). This is in line with the proposed functional differentiation of neural structures regarding fear in response to an imminent threat (defensive response) and cognitive fear/anxiety (distal, uncertain threat) whereas the latter involves more rostral cortical structures such as the mPFC and mOFC (McNaughton and Corr, 2004; LeDoux and Pine, 2016). Moreover, research on self-report measurements indicates that trait anxiety is relatively distinct from tissue damage fear, which supports a behavioral and neural dissociation of trait anxiety and PRF (Cooper et al., 2007; Perkins et al., 2007).

### Harmfulness ratings

Interestingly, although the PRF-provoking harmful activities were rated as significantly more harmful compared with the harmless activities, the ratings of perceived harmfulness during fMRI measurements were not decodable from fear-related brain response patterns. Furthermore, the ratings did not show significant correlations with PRF measures (except the PASS-F scale; Table 2). Others reported only moderate relationships (*r* values <0.39) between perceived harmfulness ratings of PHODA items and self-report measures such as the TSK, pain-catastrophizing scale, or pain intensity (Leeuw et al., 2007a), indicating that ratings of perceived harmfulness assess something akin to, but also distinct from, PRF self-report measures. The weak relationships between ratings of perceived harmfulness and self-report measures of PRF might be explained by the specificity of the potentially harmful movements depicted by the PHODA items. Namely, the ratings of perceived harmfulness were specifically related to back-straining movements such as bending and lifting, while the PRF measures might also be associated with other potentially harmful movements. As such, the fear-related neural patterns induced by the observation of potentially harmful activities for the back might not include information about movement specificity. Instead, these neural patterns might predict PRF and its constructs in a more general fashion that is captured by the TSK and FABQ.

### Limitations

A limitation of this study is the relatively small sample size in conjunction with the cross-validation framework. Ideally, the predictive model should be trained and tested with completely independent data. However, the results obtained are likely to be valid for the following several reasons: (1) the goal of the current study was not maximizing decoding performance, rather, multivariate decoding was used for the interpretation and understanding of the different PRF constructs, for which significant predictive accuracy was obtained (Hebart and Baker, 2017); (2) the applied linear SVM has been shown to exhibit good performance even in very high-dimensional settings with small sample sizes (Varoquaux and Thirion, 2014); (3) the applied regression approach using continuous variables enhances statistical power compared with a categorical analysis (e.g., low vs high fear; Altman and Royston, 2006); and (4) the variability of the regions that contribute most to the models across cross-validation folds was very small (indicated by the ER), demonstrating stable ranking, irrespective of which subject`s data were left out for validation. For these reasons, the differences of the prediction models are unlikely to be caused by the small sample size. A further limitation is related to the sparsity approach (L1 regularization) of the MKL algorithm currently implemented in PRoNTo, which does not select brain regions with highly correlated neural information. Therefore, potential lateralization effects of brain regions (e.g., left and right amygdala) should be carefully interpreted. Finally, the study design only allows interpretations of PRF to back-straining movements and LBP. Therefore, conclusions related to other musculoskeletal conditions should be drawn with caution. Nevertheless, the current approach might represent a promising new tool to dissect psychological constructs of self-report measures by using their neural predictors.

### Conclusion

This is the first time that multivariate brain response patterns have been used to better understand and dissect a psychological construct, here, PRF, conventionally assessed by self-report (questionnaires). Relating content-selective neural information to potentially different psychological constructs likely supports their construct validity by revealing (hidden) commonalities or differences across psychological constructs. Indeed, dissociable fear-related neural information served as score estimators of the FABQ (FABQ total and FABQ-W), the TSK (13- and 11-item versions), and the T-Anxiety questionnaire, supporting the distinctness of the fear constructs behind these questionnaires. The FABQ scales demonstrated strong predictive power with high sensitivity to the harmful condition and were associated with subcortical fear-processing regions (amygdala, thalamus, hippocampus). The TSKs were more related to neural response patterns of the OFC, potentially indicating that the construct of kinesiophobia is more related to higher-order brain structures associated with anxiety, while the FABQ scales are more related to subcortical defensive responses to fear. The PASS and its subscales failed to demonstrate a predictive association with fear-related brain response patterns. From a clinical point of view, it might indicate that the various PRF questionnaires, although often correlating, indeed measure different fear phenotypes related to pain. Therefore, the results emphasize the need to carefully consider the different PRF questionnaires in research and clinical settings as their constructs do not appear to be interchangeable.

## Synthesis

Reviewing Editor: Daniela Schiller, Mount Sinai School of Medicine Decisions are customarily a result of the Reviewing Editor and the peer reviewers coming together and discussing their recommendations until a consensus is reached. When revisions are invited, a fact-based synthesis statement explaining their decision and outlining what is needed to prepare a revision will be listed below. The following reviewer(s) agreed to reveal their identity: Dominik Bach. Note: If this manuscript was transferred from JNeurosci and a decision was made to accept the manuscript without peer review, a brief statement to this effect will instead be what is listed below.

This manuscript provides an interesting example of how machine learning can be used to investigate the neural mechanisms underlying psychological constructs (in this case, measures of pain-related fear), providing some interesting ideas for future work on fear of pain.

In this revision, most of the technical comments were exhaustively addressed. There are only a few points remaining.

(1) Multiple comparison correction is done by controlling for the FDR, which in itself constitutes a weak correction. This should be prominently mentioned and discussed. The rationale for including non-significant findings into the results remains unclear. The authors argue that non-significant findings are indicative of non-overlapping constructs. In this case, only presenting the cutoff values (p &lt;&gt; .05, FDR or uncorrected) is not very informative. The revised manuscript should present all FDR-corrected findings in the text, and present actual (uncorrected) p-values from all models in a systematic table.

(2) A previous comment asked the authors to give a broader picture of what we can learn for neuroscience or clinical work - the response is a bit on the weak side. The ‘distinct’ brain networks associated with different fear of pain questionnaires can only be as distinct as the questionnaires themselves. Hence, it appears that the question of distinctness could more easily be addressed with a factor analysis of the original questionnaires.
